# The effect of text message reminders on compliance with Twin Block appliances: A randomised controlled trial

**DOI:** 10.1177/14653125231188378

**Published:** 2023-08-01

**Authors:** Emily Higgins, Thérèse Garvey, Angus Burns

**Affiliations:** Orthodontic Department, Division of Public and Child Dental Health, Dublin Dental University Hospital, Trinity College Dublin, Ireland

**Keywords:** Twin Block, compliance, reminders, functional appliance, intra-oral sensor

## Abstract

**Aims:**

The aim of this study was to evaluate whether text reminders influence patient compliance with Twin Block appliances. The null hypothesis was that there was no statistically significant differences in Twin Block compliance between those who receive text reminders and those that do not.

**Design:**

Single-blind parallel randomised controlled clinical trial.

**Setting:**

Health Service Executive (HSE) orthodontic outpatient clinic in Dublin, Ireland.

**Participants:**

A total of 59 patients aged 11–15 years with a 5a Index of Orthodontic Treatment Need (IOTN grade) starting treatment with Twin Block appliances.

**Methods:**

A computer-generated unstratified allocation sequence was used to randomise the participants into the control group (CG) or the text group (TG). Both groups were asked to wear their appliances full-time. In addition to the same verbal and written instruction received by the CG, the TG received text message reminders, every 3 days, to wear their appliances. The primary outcome measure was wear time reported by Theramon^®^ sensors embedded in the appliances. Data on wear time were uploaded from the Theramon^®^ sensors onto cloud software. Participants in both groups were asked to fill out wear diaries and submit these at each visit. Treating clinicians and the primary investigator were blinded to the allocation group. Participants were followed up for 4 months. Participants were not blinded to their treatment group.

**Results:**

In total, 29 participants were allocated to the CG and 30 to the TG. The data for 53 participants were analysed, 24 from the CG and 29 from the TG. The median hours/day of wear recorded using the Theramon^®^ sensors was 13.77 (interquartile range [IQR] = 10.19) for the CG and 17.72 (IQR = 5.62) for the TG. The difference in wear time recorded was not statistically significant (*P* = 0.16).

**Conclusion:**

The study concluded that text message reminders had no statistically significant influence on patient compliance with Twin Block appliances.

## Introduction

The Twin Block appliance is a functional appliance that was first described by Clark in 1984 and later published in the *American Journal of Orthodontics* (Clark, 1988). It is the most widely used functional appliance in the UK (Chadwick et al., 1998).

### Twin Block compliance

The primary drawback of using Twin Blocks is their removable nature. A multicentre study by O’Brien et al. (2003) found a discontinuation rate of 33% with Twin Blocks used in adolescents. This reduced to 16% in the pre-adolescent group (O’Brien et al., 2003). Influences on Twin Block compliance include age, sex, duration of treatment, operator influence, patient attitude, functional and social impairments, socioeconomic status, external influence and pragmatic issues (Arreghini et al., 2017; El-Huni et al., 2019; Sinha et al., 1996; Tsomos et al., 2014; Wilson and Harris, 2015).

### Hours of wear

Previous trials comparing full- and part-time wear protocols have found no clinically significant differences between the groups (Parekh et al., 2019). In that study, however, the full-time group only wore their appliances for 12 h per day. The consensus is that the greater the wear, the greater the outcome in terms of overjet reduction. A prospective, cohort study by Arponen et al. (2020) concluded that those who wore their appliances for more than 8 h per day had an overjet reduction of 50% compared to 20% in those who wore their appliances for less than 8 h per day (Arponen et al., 2020).

### Measuring compliance

Compliance can be measured with self-reported wear reports. Self-reported wear has been shown to be unreliable, with patients, parents and orthodontists overestimating the amount of time appliances have been worn. Arponen et al. (2020) found that patients’ mean self-reported wear was 1.8 SD 5.4 h higher than actual wear time recorded by Theramon^®^ sensors (Arponen et al., 2020). Those who keep wear diaries have been shown to demonstrate more compliance with headgear than those who do not (Cureton et al., 1993). Intra-oral sensors have been shown to demonstrate reasonable accuracy in measuring wear (Charavet et al., 2019). These devices measure compliance by recording the amount of time an appliance is worn in the mouth. Studies have shown that these sensors display an acceptable level of accuracy, with Charavet et al. (2019) reporting that ‘Theramon microsensors provided an objective, reliable and accurate assessment of patient compliance’.

This reliability of Theramon^®^ sensors in recording wear time was investigated by Schott and Goz who found that Theramon^®^ had a high concordance between programmed water-bath temperature and registered wear time (Schott and Goz, 2010). This is in contradiction with a study by Brierley et al. (2017), who found that sensors that were fixed palatally under-reported wear by an average of 1.2 h per day. They concluded that increasing the range of the software temperature threshold for validating wear time would increase accuracy to more than 98% (Brierley et al., 2017).

### Improving compliance

The recommendations suggested to improve compliance include effective communication, tailoring wear regimes to individual patients, modifying appliances to suit patients’ individual needs and considering the use of reminding tools to encourage wear. The use of text messages and mobile applications has been used to enhance compliance in different aspects of healthcare (Fenerty et al., 2012). A recent Ofcom study found that 83% of 12–15-year-olds and 35% of 8–11-year-olds have their own smartphone, and this opens up opportunities for us to directly influence the compliance of these patients (Ofcom, 2019). Text message reminders are less interactive than mobile applications but have been used to encourage compliance in orthodontic patients (Eppright et al., 2014). They have the benefit of being relatively cheap and easily customised. They have been used to encourage compliance with oral hygiene, improve attendance rates, encourage elastic wear, and reduce post-treatment pain and anxiety in orthodontic patients (Bowen et al., 2015; Keith et al., 2013; Prasad and Anand, 2012; Ross et al., 2019).

The aims of this study were to evaluate the effect of text message reminders on compliance of patients with Twin Block appliances and to compare patient-reported wear times and objectively recorded wear times. The null hypotheses were that there were no statistically significant difference in Twin Block compliance between those who receive regular text message reminders and those who do not, and that there was no difference between objectively and subjectively reported wear times.

## Participants and methods

### Trial design

This was a single-blind randomised controlled clinical trial with a 1:1 allocation ratio. Ethical approval was granted by St. James’s Hospital Joint Research Ethics Committee, Dublin, Ireland (reference no.: 2020-02 Chairman’s Action 54). The initial protocol involved assessment of clinical parameters such as overjet, molar relationship and canine relationship; however, the onset of the COVID-19 pandemic mitigated against the collection of clinical data and the study protocol was altered to reflect this.

### Participants

The sample consisted of patients who were recruited from the functional appliance waiting list of the HSE Orthodontic Unit. Patients were assigned to this list after initial examination revealed an overjet greater than 9 mm, resulting in an IOTN score of 5a. They were recruited as a consecutive sample between September 2020 and November 2020. One participant refused to take part. The following inclusion criteria were applied: patients with a Class II division 1 malocclusion; patients aged 11–15 years; patients with an overjet of >9 mm; and patients with their own smartphone with the capacity to receive text messages.

Functional appliances other than Twin Blocks were excluded. Patients who had a pre-functional phase of treatment were excluded as well as those who did not own a smartphone. Patients with craniofacial syndromes were not considered for inclusion. Potential participants were given information leaflets to read outlining the study and their assent was gained before enrolment. Informed consent was gained from the patients’ guardians before their inclusion in the study. There were no stopping rules identified for this trial as Twin Block appliances are a commonly used appliance to correct anteroposterior skeletal discrepancies and the study was of short duration.

### Intervention

Standardised verbal information regarding wear protocols was provided to all participants at the start of treatment. Clinicians were asked to advise participants to start wearing their Twin Blocks as part of a full-time wear regime immediately, wearing their Twin Blocks full-time apart from sports, eating and cleaning. Participants were advised to contact the department if there were any problems with their appliances. The text group (TG) received a text message, on every third day during treatment, them to wear their appliance. Text messages were sent using a web-based Short Message Service (SMS) platform (3Communicate software, Version 12.5.18; Three Ireland (Hutchison) Limited, Sir John Rogerson’s Quay, Dublin, Ireland). These messages were sent at three different times – 09:00, 14:00 or 17:00 – and were sent directly to the participants’ phone. Treating clinicians were not given information on wear times during treatment. Treatment was carried out by five clinicians, two orthodontic registrars and three specialist orthodontists.

### Outcomes

**Figure 1.**
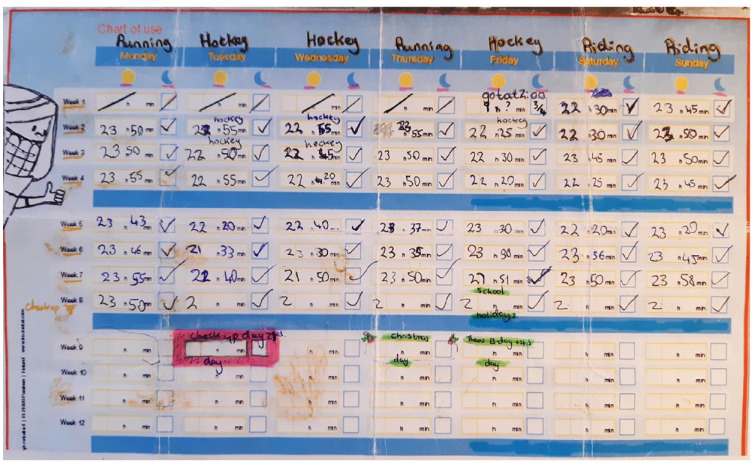
Completed wear diary.

**Figure 2.**
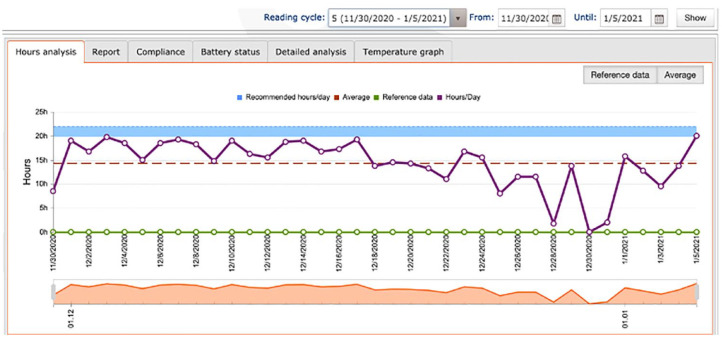
Theramon^®^ data output.

The primary outcome measure (hours of wear/day) was recorded in two ways. Participants were asked to maintain a wear diary and to bring it with them to every visit. These were to be completed once daily before bedtime to reflect the wear on that day and overnight. An example of the wear diary can be found in Figure 1. The Theramon^®^ sensor used in this study was a small (12 mm × 8 mm × 2 mm) device covered by polyurethane. It measured the temperature within the mouth every 15 min. The range of temperatures between which the appliance is deemed to be worn is 31.5°C–38.5°C and the sensor is capable of recording the temperature with an accuracy of within 0.1°C (Schott and Goz, 2010). The Theramon^®^ software read and interpreted the data (Theramon software, version 1.2.2.26; Handelsgentur, Gschladt, Austria). A diagram with the relevant information was then generated (Figure 2).

The secondary outcome measure was the number of days when more than 18 h of appliance wear was recorded. The prescribed time in this study was 18 h/day. This was to allow for removal of the appliance during mealtimes and when playing contact sports. Analysis was also carried out on the days where over 8 h of wear was recorded. This was considered as the minimum daily wear time to be considered ‘compliant’. Data for this outcome were also using Theramon^®^ data output only. Data were collected between September 2020 and May 2021.

### Sample size calculation, randomisation and blinding

The sample size calculation was carried out using G*Power 3.1.9.2 (Faul et al., 2007). A sample size calculation of 52 participants in total was determined sufficient to yield a power of 80% and an alpha of 0.05. This was calculated using a medium effect size of 0.8 based on a clinically significant difference in wear time of 4 h per day. This was based on previous studies comparing full-time and part-time wear of Twin Block appliances (Parekh et al., 2019). The recruitment of 59 patients allowed for a drop-out rate of 10% without affecting the power of the study.

A computer-generated unstratified allocation sequence (Research Randomizer; Version 4.0) was used to randomise the participants (Urbaniak and Plous, 2013). Research Randomizer (Version 4.0; http://www.randomizer.org). The sequence was kept in opaque, tamper-proof envelopes in a secure location until the allocation was required. The envelope was opened by the participant and the group revealed. Once revealed, the independent gatekeeper noted the group in a secure document that was not provided to the research lead or treating clinician. It was not possible to blind the participant to the research group. Systematic error was reduced by ensuring that the clinicians responsible for treatment were blinded as to the research group. The research group was revealed to the principal investigator before data analysis.

### Twin Block design

**Figure 3.**
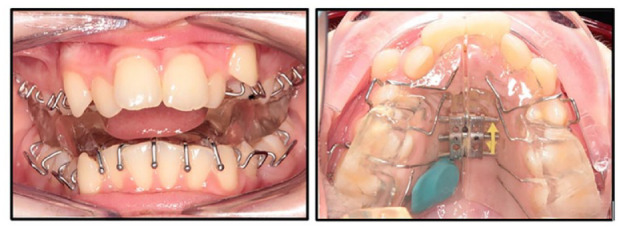
Twin Block design.

A standardised Twin Block design was used in this study (Figure 3): (1) Adams clasps on all first premolars and first permanent molars; (2) ball-ended clasps interproximally in the lower labial segment; (3) maxillary midline screw; (4) acrylic blocks intersecting at 70° with a height of 6 mm; and (5) Theramon^®^ sensor embedded in the maxillary block. Wax records were taken with participants occluding in maximum protrusion with an anterior opening of 2–3 mm. There were no cases in the study where incremental advancement was used.

### Data analysis

Data were collected between October 2020 and April 2021. Data analysis was carried out using SPSS software (SPSS Statistics for Windows, Version 25.0; IBM Corp., Armonk, NY, USA) and RStudio Team (Integrated Development for R, Version 1.4.1717; RStudio, Boston, MA, USA). A per-protocol analysis was carried out whereby incomplete data from participants who had Theramon^®^ sensor malfunction were not included in statistical analysis. The level of statistical significance was predefined at *P* < 0.05.

Theramon data: Mean hours/day of wear for both groups was determined using data from the Theramon^®^ sensor and were calculated by dividing the total number of hours of wear by the number of days the appliance was prescribed. The Shapiro–Wilk test for normality was used to determine whether the data were normally distributed. A Mann–Whitney U test was used to compare the groups as the data were not normally distributed. The number of days where more than 18 h or 8 h of wear recorded by the Theramon^®^ sensor was calculated for both the control group (CG) and TG using the data output. A negative binomial regression analysis was used to compare the groups as the data did not satisfy the criteria for a Poisson analysis.

Wear diary data: Due to the limited number of completed wear diaries returned, it was not possible to carry out a formal analysis comparing the self-reported wear in the CG and TG. Similarly, due to a large amount of missing data in the wear diary group, it was decided not to carry out a statistical analysis on objective and subjective wear times.

## Results

No harms were reported in this study. The trial continued to completion.

### Descriptive statistics

**Figure 4.**
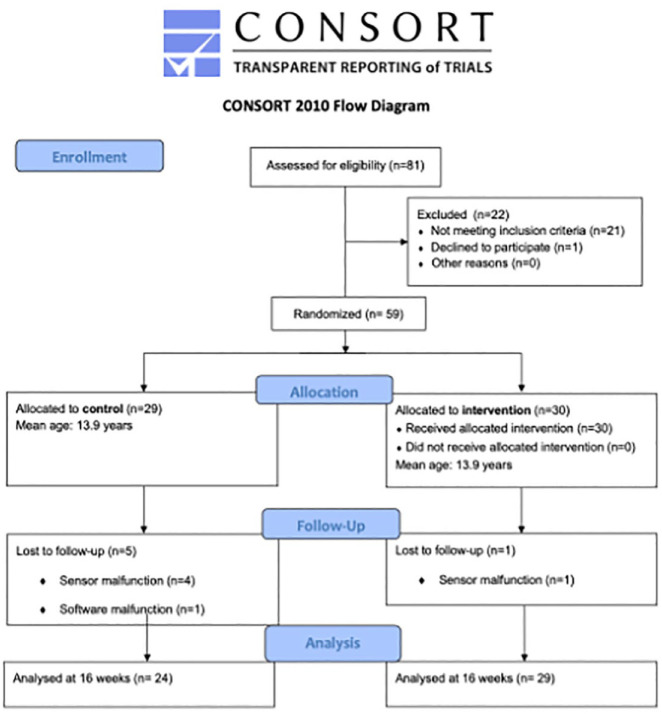
Flow of participants through the study.

The flow of participants through the study can be found on the CONSORT diagram (Figure 4). There was a total of 27 girls and 32 boys (mean age = 13.9 SD 0.57 years).

Four of the sensor batteries failed in the CG and one sensor was not possible to read due to an inability to connect to the software server as a result of a nationwide cyberattack. Therefore, data for 24 participants were analysed. In the TG, sensor battery failure rendered an incomplete dataset for one participant; therefore, the data of 29 participants were analysed. In total, the data of 53 participants were analysed.

Baseline characteristics of the participant groups are outlined in Tables 1 and 2. The groups were well matched for age at baseline with the average age of both groups being 13.9 years. The sex distribution was relatively well balanced in the CG although there was a preponderance of boys in the TG.

**Table 1.** Median age (in months) of participants.

Group	N	Median	IQR
Control	24	171.0	14
Text	29	171.0	14
Total	53	171.0	14

IQR, interquartile range.

**Table 2.** Sex distribution between the control and text groups.

Group	Sex	
	Male	Female
Text	11 (45)	13 (55)
Control	19 (65)	10 (35)
Total	30 (56)	23 (44)

Values are given as n (%).

### Objective wear time

**Figure 5.**
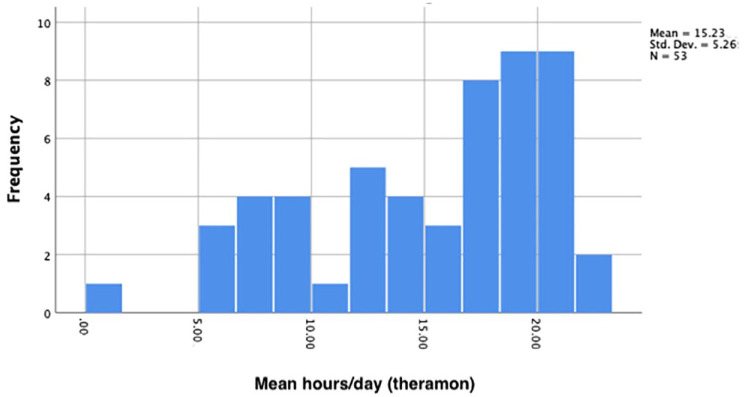
Histogram displaying non-normal distribution of data for the total sample.

The Shapiro–Wilk test was used to test for normality and this concluded that the data did not satisfy the assumption of normality (*P* = 0.21) (Figure 5). Non-parametric analysis was therefore chosen to compare the distribution of values between the groups. Table 3 illustrates the median hours of wear in the CG and TG. The Mann–Whitney U test for unpaired groups was used to test for differences in the distribution between the samples. The results demonstrated no statistically or clinically significant differences between the CG and TG (Difference 3.43 hours; *P* = 0.16).

**Table 3.** Median hours of wear for control and study groups (Theramon^®^).

Group	Control	Text	Total	Difference
Median hours/day	13.77	17.2	16.82	3.43 (*P* = 0.16)
IQR	10.19	5.62	7.59	

Gamma regression was used to determine the effect of age and sex on the average hours/day recorded in the CG and TG (Table 4).

**Table 4.** Gamma regression for average hours (Theramon^®^).

	Estimate	Standard error	95% CI	*P* value
Age	0.002	0.007	0.98 –1.01	0.76
Sex	0.01	0.1	0.83–1.23	0.85

CI, confidence interval.

### Subjective wear data

Wear diaries were returned by 28 of the sample. Of these, only five were completed in full (16 weeks of data). A total of 14 were returned with 8 weeks of data and nine were returned with 14 weeks of data. The mean subjective wear time was 18.72 SD 3.9 h. Due to the small number of completed wear diaries returned, it was not thought appropriate to complete a formal comparison between the objective and subjective hours of wear recorded.

### Compliant days

The mean number of days where more than 18 h or 8 h of wear was recorded by the Theramon^®^ sensor in the CG and TG is outlined in Table 5. In the CG, participants wore their appliances for more than 18 h a day for a mean of 43.9 SD 41.37 days and more than 8 h per day for a mean of 90.3 ± 20.14 days. In the TG, participants wore their appliances for more than 18 h a day for a mean of 61.5 ± 38.3 days and more than 8 h per day for a mean of 99.4 ± 28.77 days. Negative binomial regression concluded that the group did not have an effect on the number of days where more than 18 h or 8 h of wear was recorded (*P* = 0.26 and 0.22, respectively). The mean number of days where more than 18 h and 8 h of wear was recorded in the total sample was 58.5 ± 40.35 and 95.3 ± 24.61, respectively. Although the number of days where more than 18 h or 8 h of wear was recorded appears to be very different between the groups, the wide standard deviations atone for the lack of statistical significance.

**Table 5.** Days where more than 18 h and 8 h of wear were recorded in the CG and TG.

Parameter	Group		
	Control (h)	Text (h)	*P* value
Mean days >18 h/day	43.9 ± 41.37	90.3 ± 20.14	0.26
Mean days >8 h/day	61.5 ± 38.3	99.4 ± 28.77	0.22

Values are given as mean ± SD.

## Discussion

### Summary

The mean overall objective wear time recorded by the Theramon^®^ sensors was 15.23 SD 5.26 h. Although the participants who received text message reminders displayed an increase in mean objective wear times, these were small and not statistically or clinically significant (*P* = 0.16). Therefore, the study failed to reject the null hypothesis. The mean subjective wear time was 18.72 ± 3.9 h. This amounts to an overestimation of wear time by 3.49 h. The mean number of days where over 18 h of wear was recorded, using the Theramon^®^ sensor data, was 58.5 ± 40.35. This increased to 95.3 ± 24.61 for days where over 8 h was recorded. These figures suggest that on average, participants wore their appliances as per the prescribed regimen on just over half the days they were observed. There was no statistically significant difference in the number of days where over 18 h or 8 h of wear was recorded between the CG and TG.

### Validity and reliability

It is important that any device designed to measure wear time objectively is accurate and valid. The Theramon^®^ sensor has been shown to measure wear time accurately, judged by higher concordance between programmed water-bath temperature and registered wear time. The temperature reported by the Theramon^®^ sensor (34.5°C–35.2°C) coincided very closely to those of the water bath (35°C) (Schott and Goz, 2010). Theramon^®^ software ensures the practitioners attention is drawn to possible manipulation by registering certain temperature fluctuations as ‘unnatural’. There were five instances of battery failure in this cohort, which amounts to 8% of the sample.

### Limitations

Twin Block treatment normally occurs over a period of 9–12 months depending on patient compliance and growth. The follow-up period in this study was 4 months, which is under half of the duration of normal Twin Block treatment. This was chosen due to constrictions as a result of the COVID pandemic. Further studies may benefit from a longer follow-up, which would better establish long-term compliance.

Although there was no statistically significant difference between the CG and TG in terms of sex. There was a preponderance of boys in the TG. There is some evidence that girls display higher levels of compliance than boys (Cucalon and Smith, 1990). However, the literature is very much inconclusive, and many other studies show no difference in compliance between the two groups (Arreghini et al., 2017). The sex differences between the groups could have been minimised by carrying out a stratified randomisation.

Operator influence has been identified as an influencing factor when it comes to patient compliance. There were five treating clinicians in this study, which may have an influence on the amount of compliance recorded. A more standardised approach, assessing the patient cohort of only one clinician, would eliminate the confounding factor of operator influence on compliance. It would also improve the generalisability of the study to private practice as most patients are treated by one clinician for the duration of their treatment. In order to recruit sufficient numbers into the study in a limited time, it was decided to include multiple operators.

Clinical parameters were not influenced by wear time in a randomised controlled trial by Parekh et al. (2019). The sample size was calculated on a difference in wear of 4 h was chosen because the average wear time in the literature is approximately 12 h, and it was thought if wear was increased or decreased by 4 h or greater, there may be a clinically significant difference (Arponen et al., 2020; Parekh et al., 2019). Whether the higher wear times in this study have a clinically significant effect would be more thoroughly assessed by examining clinical outcomes of treatment rather than solely wear times.

A complete case analysis was carried out whereby incomplete data from participants who had Theramon^®^ sensor malfunction were not included in statistical analysis. Although resulting in a less biased analysis of the data, the omission of incomplete datasets reduces statistical power of the study, which increases the risk of type II error. Increasing the sample size of the study would mitigate against the risk of type II error.

### Comparison with other studies

#### Mean wear time

The mean overall objective wear time recorded by the Theramon^®^ sensors in this study was higher than that reported in the literature. The randomised controlled trial by Parekh et al. in which patients who were asked to wear their appliances for a minimum of 22 h/day wore them for only 12.38 h, or 51.6% of the time (Parekh et al., 2019). The higher recorded wear times in this study may be due to the short duration of the study, as increasing duration of treatment corresponds with a reduction in compliance. The study was carried out during the COVID-19 global pandemic and as part of the Health (Preservation and Protection and Other Emergency Measures in the Public Interest) Act 2020, restrictions were placed on Irish citizens to limit virus transmission meaning that children were unable to socialise with their peers and were obliged to remain at home for their schooling (Health (Preservation and Protection and Other Emergency Measures in the Public Interest) Act 2020, 2020). This may have reduced the external influence of peer pressure on their compliance and resulted in increased compliance in all cohorts. The mean subjective wear time was overestimated by 3.49 h. This is less than that reported by Al-Moghrabi et al. (2017), who compared patient-reported wear to objectively recorded wear found that patients over-reported their wear by 5.02 h. It is, however, in line with a study by Bos et al. (2007), who reported a difference of 3 h between patient-reported wear and wear-time measured by sensors placed in headgear straps.

#### Compliant days

Few studies are available with data regarding compliant days. A compliant day in this study was a day where the participant wore their appliance for the prescribed time (18 h/day). Analysis was also carried out on the days where over 8 h of wear was recorded. This was chosen, as the mean wear time of the part-time functional appliance group in the study by Parekh et al. was 8.78 ± 3.77 h and this group displayed similar clinical results to the full-time regimen group (Parekh et al., 2019). A study by Al-Kurwi et al. (2017) found that participants who demonstrated a wear time of more than 8 h per day had a greater overjet reduction over 5 months of treatment (Al-Kurwi et al., 2017). The mean number of days where over 18 h of wear was recorded in the total sample was 58.5 ± 40.35. This increased to 95.3 ± 24.61 for days where over 8 h was recorded. These figures suggest that on average, participants wore their appliances as per the prescribed regimen on just over half the days they were observed. There was no statistically significant difference in the number of days where over 18 h or 8 h of wear was recorded between the CG and TG (*P* = 0.26 and 0.22, respectively). With an increase in sample size, the differences may demonstrate statistical significance.

### Implications for clinical practice

The findings of this research suggest little benefit from using text message reminders to improve compliance with Twin Block wear in the first 4 months of treatment. Theramon^®^ sensors do incur an additional cost (€44/£39) and combined with the financial cost of sending repeated text messages this precludes its general use in clinical practice. It may be useful as a targeted measure for patients identified as at risk of poor compliance. Participants in this study were treated in a public outpatient department and were not paying for their treatment. Some studies have suggested that self-paying patients are less likely to be dismissed from treatment for non-compliance (Wilson and Harris, 2015); however, others have found that socioeconomic status has no effect on compliance (Mandall et al., 2008).

### Implications for research

There are few available data regarding the suitability of timing for text message reminders. Personal correspondence suggested that timing should be tailored to the patient schedule. In this instance, three different times were chosen: 09:00, 14:00 and 17:00. These were chosen as it was felt that those were times at which there was a greater risk that forgetfulness that could influence Twin Block compliance. Further research to determine patterns of non-compliance could be carried out by examining the output of Theramon^®^ sensors. Integration of the wear diary into an interactive mobile app, such as the Dental Calendar, may help improve patient interaction with the resource and result in more accurate recording of wear time. The accuracy of the Theramon^®^ sensor in its ability to record wear time and the user-friendly cloud interface makes it useful tool for objectively measuring compliance. Future research examining the compliance of patients with removable and functional appliances would benefit from the use of objective measuring devices, such as intra-oral sensors. Furthermore, future studies should examine the effect of reminders factors such as clinical outcome, overall treatment time and patient-reported outcomes.

## Conclusion

The following conclusions can be drawn from this investigation: (1) text message reminders have no statistically or clinically significant influence on patient compliance with Twin Block appliances in the first 4 months of treatment; and (2) text message reminders have no statistically significant influence on the number of days where over 18 h or over 8 h of wear is recorded.

## Supplemental Material

sj-pdf-1-joo-10.1177_14653125231188378 – Supplemental material for The effect of text message reminders on compliance with Twin Block appliances: A randomised controlled trial Supplemental material, sj-pdf-1-joo-10.1177_14653125231188378 for The effect of text message reminders on compliance with Twin Block appliances: A randomised controlled trial by Emily Higgins, Thérèse Garvey and Angus Burns in Journal of Orthodontics
